# Knockdown of Broad-Complex Gene Expression of *Bombyx mori* by Oligopyrrole Carboxamides Enhances Silk Production

**DOI:** 10.1038/s41598-017-00653-3

**Published:** 2017-04-11

**Authors:** Asfa Ali, Venugopal Reddy Bovilla, Danti Kumari Mysarla, Prasanthi Siripurapu, Rashmi U. Pathak, Bhakti Basu, Anitha Mamillapalli, Santanu Bhattacharya

**Affiliations:** 1 Department of Organic Chemistry, Indian Institute of Science, Bangalore, 560 012 India; 2 Director’s Research Unit, and Technical Research Centre, Indian Association for the Cultivation of Science, Jadavpur, Kolkata, West Bengal 700 032 India; 3 Department of Biotechnology, GITAM Institute of Science, GITAM University, Visakhapatnam, 530 045 India; 4 Centre for Cellular and Molecular Biology, Hyderabad, 500 007 India; 5 Molecular Biology Division, Bhabha Atomic Research Centre, Mumbai, 400 085 India

## Abstract

*Bombyx mori* (*B*. *mori*) is important due to its major role in the silk production. Though DNA binding ligands often influence gene expression, no attempt has been made to exploit their use in sericulture. The telomeric heterochromatin of *B*. *mori* is enriched with 5′-TTAGG-3′ sequences. These sequences were also found to be present in several genes in the euchromatic regions. We examined three synthetic oligopyrrole carboxamides that target 5′-TTAGG-3′ sequences in controlling the gene expression in *B*. *mori*. The ligands did not show any defect or feeding difference in the larval stage, crucial for silk production. The ligands caused silencing of various isoforms of the broad-complex transcription factor and cuticle proteins which resulted in late pupal developmental defects. Furthermore, treatment with such drugs resulted in statistically enhanced cocoon weight, shell weight, and silk yield. This study shows for the first time use of oligopyrrole carboxamide drugs in controlling gene expression in *B. mori* and their long term use in enhancing silk production.

## Introduction

The domesticated silkworm*, Bombyx mori* (*B. mori*) is a lepidopteran model with four developmental stages, namely egg, larva (caterpillar), pupa, and adult. The silk glands produce large amounts of silk proteins during the final stage of the larval development. The development of silk gland and its growth depends on various factors such as environment, rearing method, and leaf quality etc. Among all the factors, the nutrient value of the mulberry leaf and environmental conditions influence the silk production^1, 2^. Since cocoon is the final crop yield, care must be taken for its better and healthy spinning by feeding the worms with good quality leaves. It has been observed that silkworms obtain 72–86% of their amino acids from mulberry leaves and more than 60% of the absorbed amino acids are used for silk production^3^.

In eukaryotic nucleus, satellite DNA (satDNA) forms an integral part of the heterochromatin and is characterized by tandemly repeating sequences^4^. The oligodeoxynucleotide (ODN) stretch 5′-TTAGG-3′ is the most conserved satellite repeat in most of the insect species. This repeat is present in all species of order Lepidoptera, Hymenoptera, Trichoptera, Mecoptera, and also in other orders. However, the 5′-TTAGG-3′ repeat is absent among all species of the order Diptera^5–7^.

Specific targeting of insect and vertebrate telomeres with pyrrole and imidazole polyamides opened up sequences in the telomeres and showed the importance of these repeats^8, 9^. These studies also showed the protein binding nature of the satellite repeats^10^. These drugs bind to specific DNA sequences with remarkable affinities^11–13^. While various oligoamide drugs bind to human 5′-TTCCA-3′^14^, 5′-GAGAA-3′ of *Drosophila* ^12^ etc., few altered the transcription by targeting 5′-GAA-3′.3′-TTC-5′^15^. Apart from targeting the drugs to heterochromatic regions, pyrrole based drugs were also used to control the expression of genes^16^. Certain ligands with greater specificity toward DNA sequences, control their expression better than the transcription factors^17^. It has also been reported that several pyrrole polyamide drugs can be used to downregulate gene expression^18^. Similar observation is reported with matrix metalloproteinase (MMP)-9 gene expression as well^19^. Recently, it was shown that *p*-chloro, *p*-bromo, and *p*-azido-substituted *L*-phenylalanine incorporation into the silk fibroin by *in vivo* feeding of the transgenic silkworm resulted in a decrease in the production of fibroin^20^. Tandem repeat sequences were found to be enriched in the promoter region of genes in *Saccaharomyces cerevisiae* ^21^. The repeat number in the promoter region was shown to be important in regulation of gene expression in *Neisseria meningitidis* ^22^.

Our interest stems from the potential use of such ligands in controlling gene expression^23, 24^ and their application in seribiotechnology. Herein, we report for the first time, genome targeting of *B*. *mori* with 5′-TTAGG-3′ interacting oligopyrrole carboxamides and show the knockdown of broad-complex and cuticle gene expression. Moreover, it was found that these drugs also resulted in the enhancement of silk production, a finding which may have significance in sericulture.

## Results

### Synthesis of dimeric oligopyrrole carboxamides

**Figure 1:**
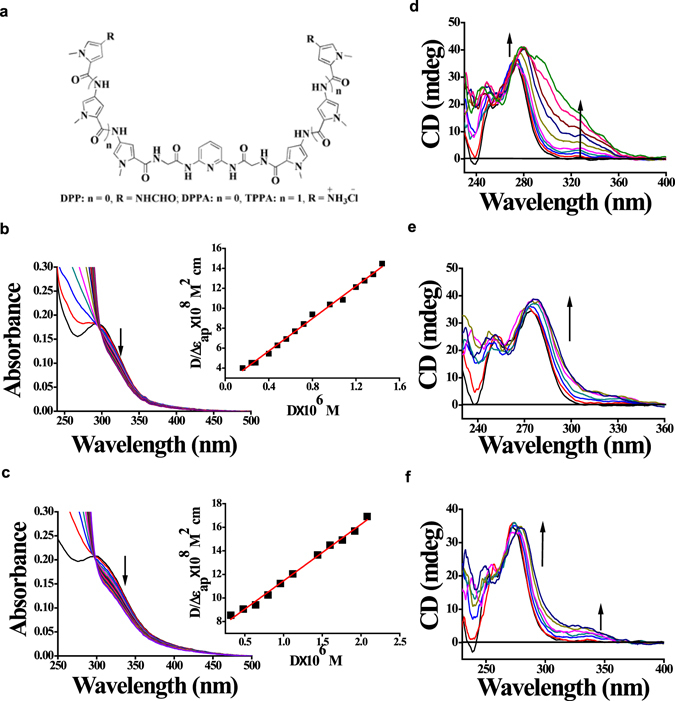
Binding data of various drugs to 5′-TTAGG-3′ repeats. (**a**) Molecular structures of **DPP**, **DPPA** and **TPPA**. UV-Vis absorption spectral titrations of 20 μM of (**b**) **DPPA** and (**c**) **TPPA** with increasing concentration of *Bm*-dup_20_ with half-reciprocal plots as inset. CD titrations of *Bm*-dup_20_ were performed with increasing aliquots of (**d**) **DPP** (**e**) **DPPA** and (**f**) **TPPA** in sodium phosphate (20 mM), NaCl (20 mM) buffer (pH 7.0) at 25 °C.

In order to target the 5′-TTAGG-3′ sequence, we have synthesized novel oligopyrrole carboxamide ligands (**DPP**, **DPPA**, and **TPPA**) (Fig. 1a). 2, 6-Diaminopyridine was converted to 2-amino-N-[6-(2-aminoacetamido)pyridin-2-yl] acetamide (**SI-7**) which was then reacted with appropriate oligopyrrole carboxylic acids to yield the corresponding dimeric oligo-4-nitropyrrole carboxamides (**SI-10**, **SI-11**)^23^. These were finally reduced over H_2_-Pd/C followed by the addition of formic acid or dil. HCl to yield the products-**DPP**, **DPPA**, and **TPPA**, respectively (see Supporting Scheme S1). While **DPP** possesses terminal formamide groups, **DPPA** and **TPPA** end with free ammonium groups.

### Biophysical parameters of ligand interaction with ODNs

Absorption spectral titrations revealed interaction of the ligands with [poly(dA-dT)]_2_ but not with [poly(dG-dC)]_2_ (Supplementary Fig. 1). A substantial hypochromism was observed for each ligand with the pre-formed d[(5′-(TTAGG)_4_-3′)/(3′-(AATCC)_4_-5′)] duplex DNA (*Bm*-dup_20_). This further indicates more efficient binding to *Bm*-dup_20_ by **DPPA** and **DPP** compared to **TPPA** (Fig. 1b, Supplementary Fig. 2, Fig. 1c, Supplementary Table 1). Furthermore, circular dichroism (CD) spectral studies revealed significant affinity of **DPPA** and **DPP** toward the duplex DNA as confirmed by the increase in the molar ellipticity at 272 nm peak and a noticeable inversion was observed in the molar ellipticty at the negative peak (239 nm) upon interaction with the ligand (Fig. 1d–f). The appearance of the induced CD (ICD) bands at ~320–340 nm, followed by saturation of the peak at ligand:DNA ratio ranging ~3.5–5, emphasizes the interaction of the ligands with the chiral grooves of the duplex DNA.

### Drugs do not show any effect on the nuclear localization of telomeric 5′-TTAGG-3′

**Figure 2:**
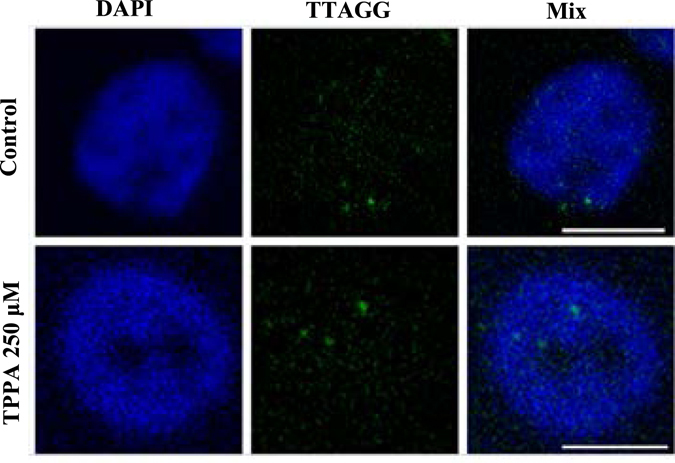
DNA-FISH of control and drug treated wing discs. The drug treated and control wing disc nuclei show condensed 5′-TTAGG-3′ satellite DNA. No change was detected in the number of TTAGG signals observed between control and drug treated nuclei. DNA was visualized by DAPI staining. Scale bar, 10 µm.

Since the 5′-TTAGG-3′ sequences are predominantly present at the heterochromatic regions of the telomere in *B*. *mori*, we tested the changes induced by the drug at such regions. DNA-FISH experiments were performed with wing discs of control and TPPA treated larvae of *B. mori* on day 6 of their fifth instar stage. In both the wing disc nuclei of control and drug treated larvae, 5′-TTAGG-3′ signals were found as bright condensed spots signifying the heterochromatic regions (Fig. 2). A total of ninety four nuclei were counted in three control wing discs. The number of spots ranged from 1–9 per nucleus. The average number of spots per nucleus was found to be 4.38. In the wing discs of drug treated larvae, TTAGG signals were also found to be condensed. Ninety nine nuclei from five wing discs were counted. The number of spots ranged from 2–9 per nucleus and the average number of signals per nucleus was found to be 4.58. The nuclear DNA stain, DAPI, did not show any difference in staining between the wing disc nuclei of control and drug treated larvae. No gross change was observed of 5′-TTAGG-3′ distribution at the heterochromatic regions in the in the nuclei of control and the drug treated worms.

### Effect of the drug on the development of *B. mori*

**Figure 3:**
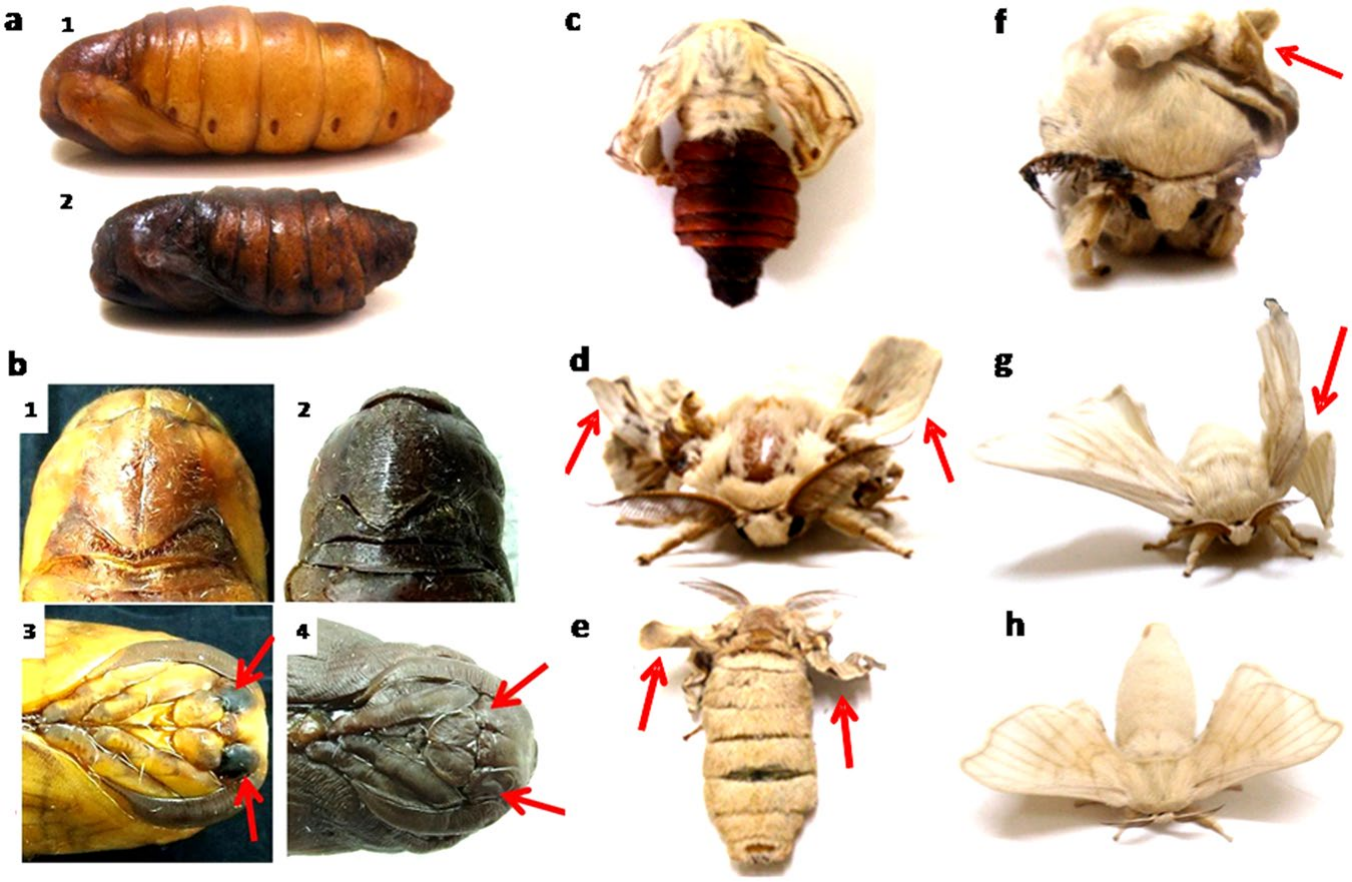
Ligand induced adult-pupal arrest with defects at pupal and adult stage. (**a**) (1) Control pupa (2) Pupa with 250 μM of **DPPA**. (**b**) Front and dorsal view of head region of pupa (1, 3) control and (2, 4) with 250 μM of **DPPA**. Arrowheads indicate proper eye in (3) and no eye in (4). (**c**) Half-fly pupal-adult arrest with 250 μM of **DPP**. Wing deformity (shown with arrowheads) in adult flies treated with (**d**,**e**) 250 μM of **DPP** (front and dorsal view, respectively) (**f**) 250 μM of **TPPA** and (**g**) 250 μM of **DPPA**. (**h**) Normal wings in control adult fly. The photographs have been taken by one of the coauthors, AA.

The biophysical analyses showed evidence of significant interaction of the drugs with 5′-TTAGG-3′ sequences. Therefore, we proceeded to evaluate their effect on the developmental parameters of the silkworm. A comprehensive study was carried out where the silkworms were treated on day 1 of the fifth instar stage with a range of the drug concentrations. The drug fed larvae neither exhibited any visible abnormality in the larval stage nor did we observe any feeding difference among the various treated and control groups post treatment. No larval/pupal arrest was seen, although defects were observed in the late pupal stage. The ligands caused ~8% pupal defects where the formation of the head and eyes were improper and faulty (Fig. 3a,b). Interestingly, we observed ligand induced stagnation of metamorphosis from pupa to adult which is clearly revealed by the half-fly image (Fig. 3c). All ligands exhibited several phenotypic aberrations during the pupal-adult metamorphosis which predominated at higher concentrations (250 µM). The emergence of the flies was impeded with >15% pupal-adult arrest for **DPP** and **DPPA** while >14% adult flies which emanated, exhibited severe abnormality in the wings in the case of **DPPA** and **TPPA** (Fig. 3d–h, Supplementary Table 2). All ligands induced similar impairments but with varying intensities, suggesting that similar cellular mechanisms might have been affected. Sericulture necessitates killing of the pupae by boiling to avoid the damage of the cocoons by the emerging flies. Here, the late pupal and adult defects and/or lethality may be advantageous since it may diminish the spoilage of the cocoon, and hence would potentially benefit silk production at the farm level.

### *In silico* analysis of 5′-TTAGG-3′ and expression analysis of 5′-TTAGG-3′ enriched genes

**Figure 4:**
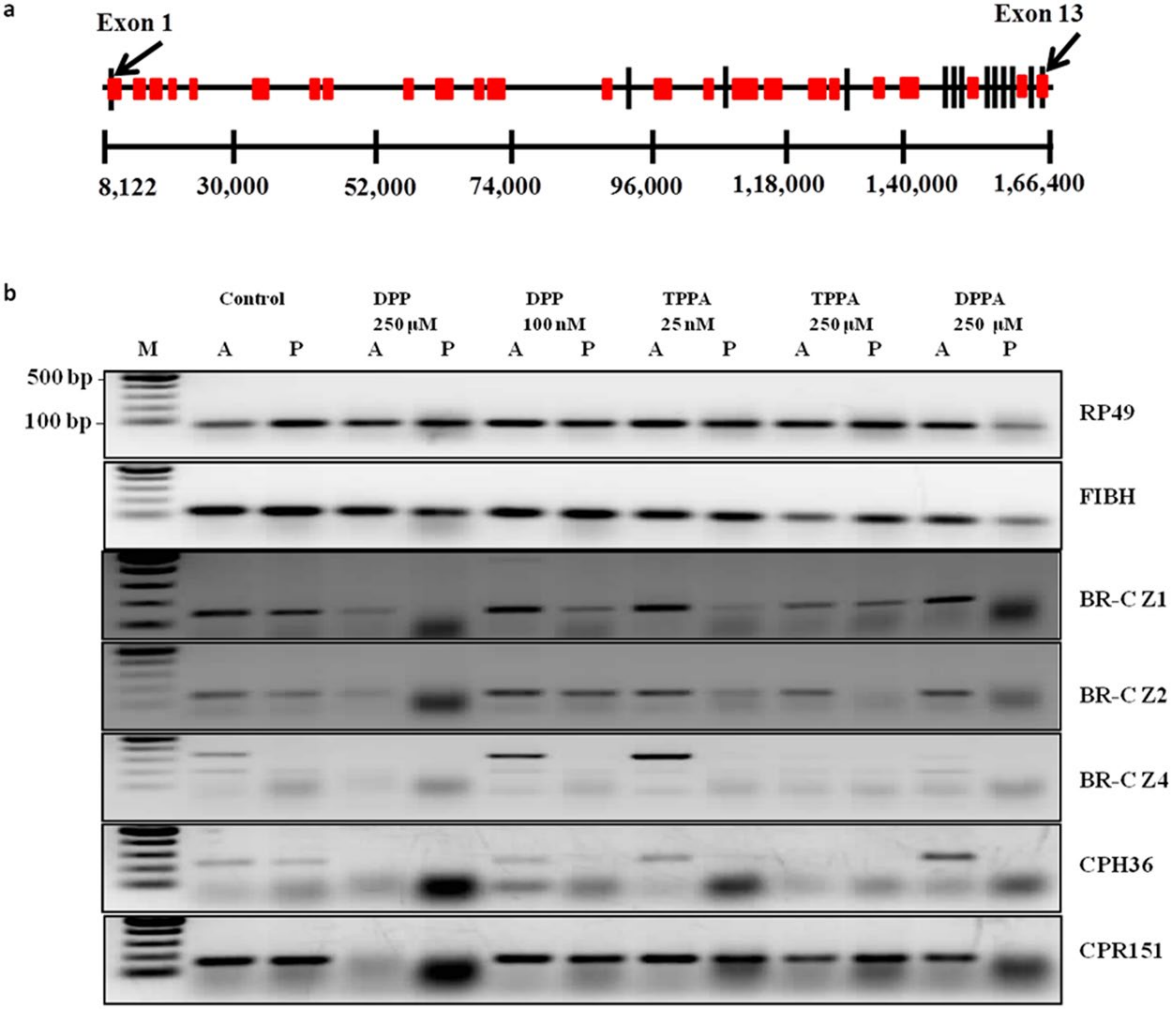
TTAGG enriched BR-C gene and its transcription along with other TTAGG genes. (**a**) A schematic representation of BR-C gene. The exons and introns are indicated. The red boxes indicate 5′-TTAGG-3′ repeats. Lines connect the actual order of the exons. TTAGG repeats are not to scale. (**b**) RT-PCR data for expression of RP49, FIBH, broad-complex isoforms (BR-C Z1, BR-C Z2, BR-C Z4) and putative cuticle protein genes (CPH36 and CPR151). ‘A’ and ‘P’ denote anterior and posterior silk gland.

Although 5′-TTAGG-3′ repeats are present abundantly at the telomere^5^, short stretches of such repeat sequences are found in the euchromatic regions. Moreover, as the developmental defects, wing and eye, matched with the RNAi knockdown experiments of the broad-complex (BR-C) gene, we performed BLAST analysis for finding out the occurrence of the repeats in the genome of *B. mori*. The BLAST analysis showed high occurrence of the repeats throughout the BR-C gene (Fig. 4a), especially in the 5′-UTR (untranslated region) of exon 1 from nt 8164–8177. As the 5′-TTAGG-3′ repeats span the BR-C gene, we checked the effect of targeting the 5′-TTAGG-3′ sequences on the expression of broad-complex gene. RT-PCR analysis of the control and drug treated silk glands was carried out. Fifth instar larvae of *B. mori* were treated with various concentrations of drugs, **DPP**, **DPPA**, and **TPPA**. Larvae of the fifth instar stage were sacrificed on day 6 and silk glands from the control and drug treated worms were used for RNA isolation. The dissected silk glands were segregated into anterior and posterior regions for expression analysis. RT-PCR analysis of the samples (silk glands) from the control and drug treated worms was performed with different isoforms of the BR-C genes (Fig. 4b). Knockdown of the Z1 isoform (BR-C Z1) was found in the posterior region upon treatment with **DPP** (250 µM) and **DPPA** (250 µM). The anterior region did not show any knockdown effect. However, the control glands showed amplification of the BR-C Z1 both in the anterior and posterior regions. Broad-complex Z2 isoform (BR-C Z2) was downregulated with all ligands (250 µM) in the posterior silk gland. The impact of the drugs on the Z4 isoform (BR-C Z4) expression was quite pronounced. While the control gland showed facile expression of BR-C Z4, lower concentration of the ligands [**TPPA** (25 nM) and **DPP** (100 nM)] actively silenced the gene expression along with higher concentration of the drugs.

Besides, in other genes, namely the cuticle genes, CPH36 and CPR151, primarily in the intronic regions a stretch of 12–15 base-pairs in the form of 5′-TTAGG-3′ repeats are distributed (Supplementary Table 3). Cuticle gene, CPR151 gene, was found to possess a single stretch of 15 nt of 5′-TTAGG-3′ from 3372–3386 in the intronic region. The cuticle genes have been identified in the silk glands of *B. mori*. Cuticular proteins constitute ~25% of the total anterior silk gland proteome^24^. They are known to play a significant role in providing a favorable physiological environment for the silk fiber formation^25^. The major feature of the *Bombyx mori* anterior silk gland (ASG) cuticle proteins is that they are endowed with a typical chitin-binding domain. It is assumed that the chitin lining in the ASG, like the spinning duct of spider, act as a dialysis membrane responsible for the exchange of water and ions along the duct^26^.

The expression of CPR151 in the silk glands of the control and drug treated worms was checked since EST database showed the expression of some cuticle genes in the silk gland during the larval developmental stage. While **DPP** (250 µM) downregulated the CPR151 expression in both anterior and posterior silk glands, knockdown was witnessed only in the posterior region with **DPPA** (250 µM). The other concentrations did not show any effect on the transcription levels of this gene. More pronounced effect was found in the CPH36 which has a single stretch of 12 nt 5′-TTAGG-3′ from 3722–3733 and it has been found to be silenced in most of the treatments (Fig. 4b). The cuticular genes (CPR151 and CPH36), having intronic 5′-TTAGG-3′ sequence, showed downregulation in gene expression. It could also be possible that these genes might be regulated by BR-C which is an early transcription factor and many genes are known as the downstream targets of this complex^27–32^.

The BLAST results showed the presence of 5′-TTAGGTTATGTTAGG-3′ sequence at position 4671–4687 in the 5′-promoter region of fibroin heavy chain gene (FIBH). In the promoter region, the sequence from −1659 to −1590 plays a critical role in the promoter activity^33^. Since the 5′-TTAGG-3′ sequence is not present in the core promoter region, we did not expect any change in the expression of fibroin gene after drug treatment. Interestingly, FIBH expression remained unaltered in both anterior and posterior regions of silk glands from all treatment groups, and at all tested concentrations of the drugs.

The specific knockdown of BR-C gene and cuticular genes expression having 5′-TTAGG-3′ sequences may be correlated to the developmental defects observed *in vivo* after drug treatment and also with the biophysical data. The ligands inhibited the expression of BR-C, CPH36, and CPR151 genes which have 5′-TTAGG-3′ repeats in their 5′-UTR region or in the intronic region. However, the FIBH gene which had 5′-TTAGG-3′ at a distant position on its promoter did not show any noticeable change in its expression. The control primers used in the study, RP49 (ribosomal protein) did not show any variation in the treated samples. In order to check if the knockdown effect of the broad gene was time-dependent, posterior silk glands isolated from **DPP** (250 µM) treated and control worms were checked for the expression of RP49, FIBH, CPH36, and BR-C complex during fifth instar larval and pre-pupal stages (days 5, 7, and 9). The results showed that the knockdown effect was specific to BR-C complex and the effect was seen on all the days (see Supplementary Fig. 3). Also, BR-C has been found to be an upstream regulator of cuticle genes in *Bombyx mori* ^34^. Accordingly, the cuticle gene, CPH36, also showed knock-down effect on all the days similar to that shown by the various BR-C isoforms.

### Proteomic studies of control and drug treated silk glands

**Figure 5:**
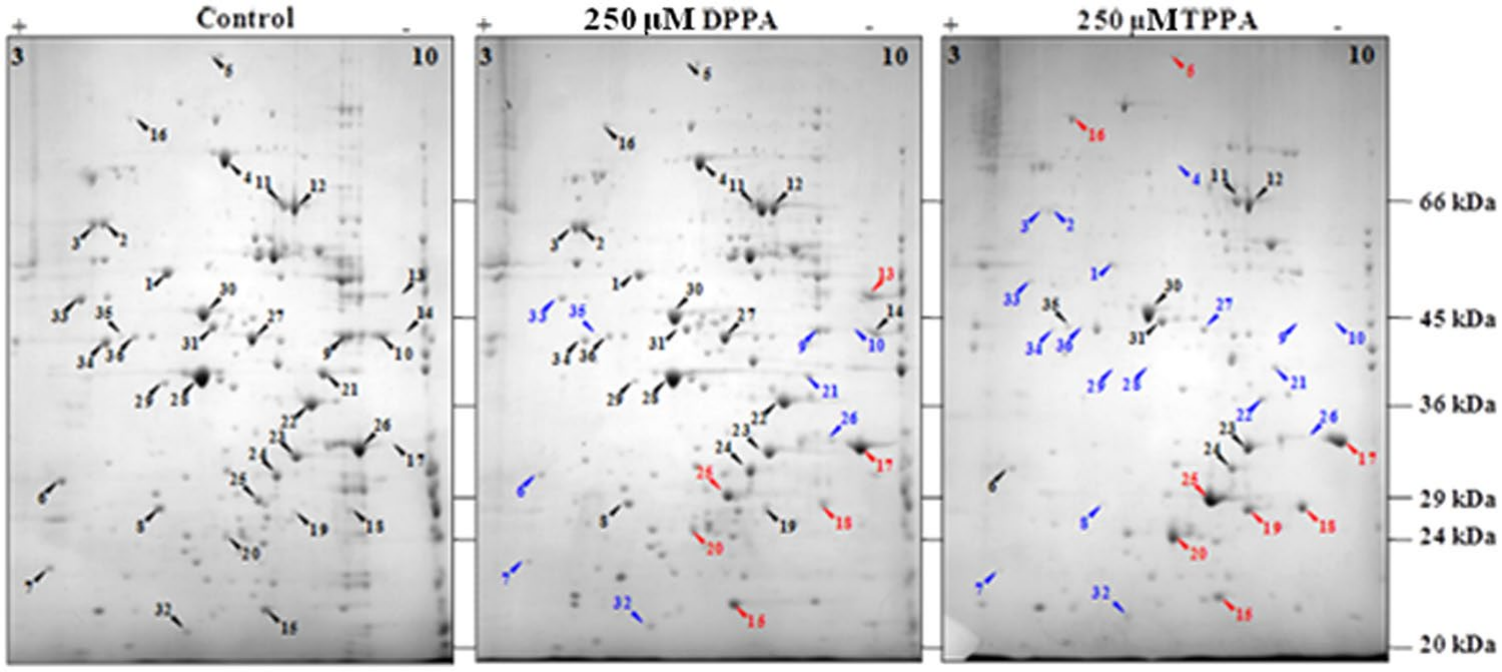
2D gel images indicating whole cell protein profile of the control or drug treated silk glands. Constitutively expressed, or induced, or repressed proteins are indicated by black, red, or blue arrow heads, respectively, and by numbers as given in the Supplementary Table 4. Molecular mass (kDa) and pI are shown on right-hand side and on top, respectively.

To understand the effectors of BR-C knockdown mediated delayed silk gland degradation phenotype, cellular proteins of the silk glands from control or the drug treated worms were resolved by two-dimensional gel electrophoresis and differentially expressed proteins were identified by mass spectrometry (Supplementary Table 4). **DPPA** or **TPPA** treated silk glands displayed increase in abundance of 6 or 8 proteins, respectively, compared to control silk glands (Fig. 5). Remarkably, increased level of 4 proteins was common to the proteins from silk glands of the worms that were treated with both the drugs. These proteins were lipoprotein 7 (spot no. 18), lipoprotein 21G1 (spot no. 20), lipoprotein 11 (spot no. 25), and thiol peroxiredoxin (spot no. 15). Incidentally, all the three identified lipoproteins and thiol peroxiredoxin are antioxidant proteins having anti-apoptotic functions^35–39^. Cellular detoxification plays an important role in silk gland development^40^. Peroxiredoxins are proteins which protect tissues/cells from oxidative damage. These proteins have been shown to be important in preserving homeostasis and extending life span in *Drosophila* ^41^. Thiol peroxiredoxin, identified in silk gland^42^, acts as a physiological anti-oxidant^43^ and helps in stress and immune response^24^. Their increased abundance has a perfect correlation to delayed silk gland degradation observed in drug treated silk glands resulting in enhanced silk production. Some of the lipoproteins and thiol peroxiredoxin have been involved in antiviral and antifungal defense system of the organism, respectively^44^. Direct regulation of expression of thiol peroxiredoxin or 30-kDa lipoproteins by BR-C genes is not known. Thus, the drugs induce oxidative stress which in turn trigger the antioxidant enzymes and result in the induction of lipoproteins which are known to involve in anti-oxidant properties. An increase in the glyceraldehyde-3-phosphate dehydrogenase (spot no. 17, basic pI), with concomitant equivalent decrease in glyceraldehyde-3-phosphate dehydrogenase (spot no. 26, acidic pI), indicate possible inhibition of phosphorylation. The reduction in the levels of 9 or 18 proteins was also observed in the silk glands of **DPPA** or **TPPA** treated worms. The observed proteomics profile could be due to the overall complex network of interactions as BR-C showed eleven physical and two genetic interactors in *Drosophila* (BioGRID)^45^.

### Economic parameters analysis after drug feeding

**Figure 6:**
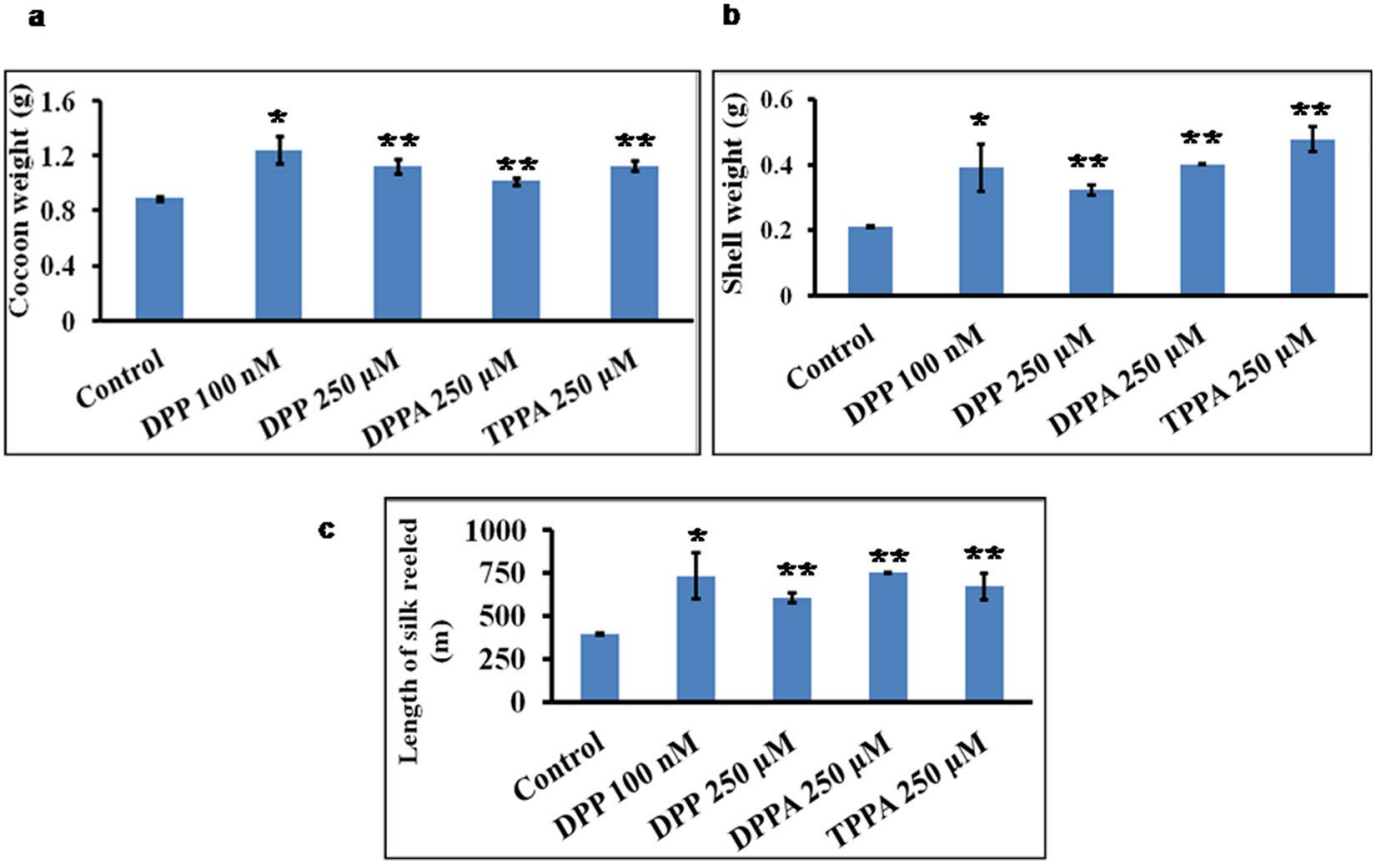
Effect of **DPP**, **DPPA**, and **TPPA** on various growth parameters related to silk economics; (**a**) the cocoon weight (**b**) the shell weight and (**c**) the length of silk reeled. Biological replicates of control and drug treated groups were conducted each time. The data of one biological replicate (control and different drug treated groups) was analysed by one way ANOVA and student’s *t*-test (Two-Sample Assuming Equal Variances). The bars labeled with ‘*’ are significantly different (*p* < 0.05) and labeled with ‘**’ have *p* < 0.01.

Silkworms are commercially reared for producing silk. We, therefore, examined the effect of the drug on the economic parameters. The silkworms were treated on day 1 of the fifth instar stage with a range of drug concentrations and parameters like the cocoon weight, shell weight, and length of the silk reeled were analyzed. The results showed an increase in the cocoon weight (Fig. 6a), shell weight (Fig. 6b), and hence increase in the length of the silk reeled (Fig. 6c). However, there were variations in outcome among different drugs and their concentrations. In comparison to the control group, all treated groups showed increase in cocoon weight, shell weight, and length of silk reeled. **DPP** showed greater enhancement in the economic parameters at low concentration but with high error values. The treated groups of **DPP** (250 µM), **DPPA** (250 µM), and **TPPA** (250 µM) showed differences in the cocoon weight, shell weight, and length of the silk yield. **DPPA** (250 µM) treated larvae showed consistent economic parameters in all treatments and significant increase in the length of the silk reeled in comparison to control with very low error values.

## Discussion

This study reports for the first time the use of oligopyrrole carboxamides in the regulation of broad-complex and cuticle gene expression in *B. mori*. The synthesized drugs **DPP**, **DPPA**, and **TPPA** could efficiently knockdown the broad-complex and cuticle gene expression. The degree and extent of knockdown varied with the drug, the concentration of the drugs used and the location of 5′-TTAGG-3′. The leading amide unit at the termini of **DPP** appears to exert additional hydrogen bonding interactions with the DNA which is absent in the case of **DPPA**. As a result, **DPP** may have induced gene knockdown at a lower concentration. However, at a higher concentration, longer oligoamide, **TPPA**, did not interact as efficiently as **DPPA** which emphasizes the requirement of length matching of the N-methylpyrrole oligomers for their optimal binding with the duplex DNA. The cell biology experiments with the wing discs of control and drug treated worms did not show any change in the localization of 5′-TTAGG-3′ heterochromatic regions, so we looked for the presence of 5′-TTAGG-3′ in the gene rich euchromatic regions.

The BLAST analysis showed the presence of 5′-TTAGG-3′ repeats in the BR-C, cuticle, and fibroin genes. Knockdown of BR-C gene after drug treatments showed the *in vivo* specificity of the drugs in interacting with 5′-TTAGG-3′ sequence. The enhanced efficiency of the drugs in interacting with the BR-C transcription factor has been manifested with various defects at the late pupal and adult stages of *B. mori*. Moreover, the phenotypic (wing and eye) defects observed in the present study matched with the BR-C knockdown by RNAi^46^. Though the drugs had target sites in the *B. mori* heterochromatin and euchromatin genome, matching of developmental defects observed proves broad-complex gene as the major target of the drug. The fibroin gene which has single or two times 5′-TTAGG-3′ repeats are unaffected by the drug treatment, shows the requirement of optimum repeat length for the drug to bind. The observed knockdown of cuticle gene expression could be due to the direct interaction of the drug as these genes have 5′-TTAGG-3′ repeats or could also be due to the knockdown of broad-complex as there are downstream targets of the broad-complex gene. *Drosophila rbp* mutants, devoid of BR-C Z1 isoform, retained their salivary glands even 12–22 h after pupa formation^46^. Hence, knockdown of BR-C Z1 may have caused the silk glands to remain for a longer time. However, incomplete knockdown of BR-C eventually led to the silk gland degradation.

Moreover, the overall protein changes observed shows either direct effect observed due to the drug interaction with the 5′-TTAGG-3′ repeats or they could as well be due to the depletion of broad-complex as it is an early transcription factor with complex network of interactions^45^.

**Figure 7:**
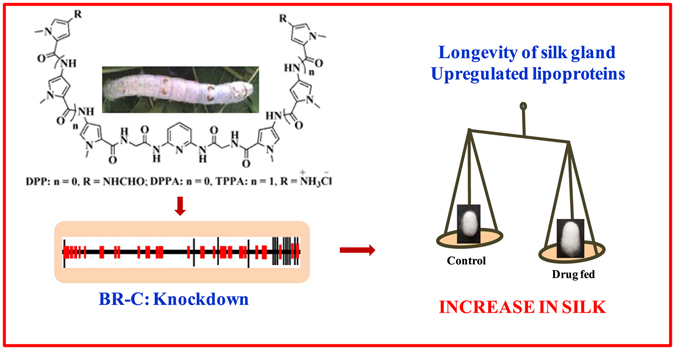
Effect of drugs on knockdown of broad-complex gene leading to enhanced silk production. The figure shown here is a compilation of images. The photographs of worm and cocoon were taken by one of the coauthors, AA. Compound molecular structures and the schematic representation of BR-C gene were drawn by AA. The red boxes indicate 5′-TTAGG-3′ repeats. Lines connect the actual order of the exons. The TTAGG repeats are not to scale. The weighing balance was drawn by AM and AA.

The increase in silk gland weight and shell weight after drug treatment is surprising but not an unexpected result. Natural polyamines bind to the DNA^47^. Polyamines like spermidine and spermine are also known to promote growth^48^ or influence gene expression^49^. Macrocyclic and acyclic polyamines in micromolar concentrations resulted in the increase of lamellopodia growth within minutes after administration^50^. Targeting of 5′-AAGAG-3′ repeats by RNAi in *Drosophila* showed an increase in the size of the pupa with late pupal lethality^51^. The use of small moleucles to target a protein and inhibit its function was earlier exploited in malaria^52^. The present work demonstrates a new approach which mediates via small molecule based targeting of 5′-TTAGG-3′ repeats of broad-complex gene to control its expression. It appears that the knockdown of the broad-complex delays the silk gland degradation, increases silk gland weight along with the upregulation of lipoproteins. Taken together, this results in significantly enhanced silk production (Fig. 7). To our knowledge, the observed enhancement in silk yield is not reported by any other approaches such as breeding^53^, transgenics^54^, and other strategies^55^ so far tested in *B. mori*. The results obtained, therefore, show a great promise in the usage of this class of drugs both for controlling the gene expression and for enhancing the yield in sericulture.

## Methods

### Chemical Synthesis

All syntheses were performed according to Supplementary Methods. The NMR spectra (^1^H NMR and ^13^C NMR) of all the ligands were shown in Supplementary Figs 4–17.

### Duplex DNA formation

Two 20-mer oligonucleotides d(5′-(TTAGG)_4_-3′) and d(5′-(CCTAA)_4_-3′) were mixed and incubated in a solution containing 20 mM sodium phosphate, 20 mM NaCl maintained at pH 7.0. It was heated at 95 °C for 5 min followed by slow cooling to 25 °C over a period of 24 h to form d[(5′-(TTAGG)_4_-3′)/(3′-(AATCC)_4_-5′)] duplex DNA (*Bm*-dup_20_).

### UV-Visible spectral titration

A stock solution of 20 μM base-pair concentration of *Bm*-dup_20_ and 200 μM base-pair concentration of [poly(dA-dT)]_2_ and [poly(dG-dC)]_2_ were used for the titrations. DNA aliquots from the stock solutions were added progressively to the ligand solution (10–20 μM) in the same buffer and continued until saturation was obtained. The intrinsic binding constants were determined by the half-reciprocal plot method^56^.

### Circular Dichroism Spectroscopy

The preformed duplex DNA (*Bm*-dup_20_) (20 μM base-pair concentration) in 20 mM sodium phosphate, 20 mM NaCl, pH 7.0 buffer was taken to which each drug solution was added gradually in a 1 cm quartz cuvette maintained at 25 °C. After an incubation time of 15 min, the CD spectra were recorded at a scan rate of 50 nm/min.

### Feeding of the worms

Fifth instar larvae of *B. mori* were fed with fresh mulberry leaves coated with ligands on the first day for a period of 24 h. A set of 30 larvae were taken for each treatment. They were nurtured with normal leaves thereafter and dissected on the sixth day as they reached the wandering stage after dipping them in Insect Ringer’s solution (0.68% aqueous NaCl solution). Several concentrations (10 nM–500 μM) were used to screen the efficacy of the drugs for growth and standardization of other parameters. Based on the increase in larval body weight and silk gland weight, we narrowed down the concentration for the tested drugs. The treatments were repeated five times and each time, the parameters were measured with respect to the control kept at the same environmental condition but fed with normal mulberry leaves.

### DNA fluorescence ***in situ*** hybridization (DNA-FISH)

DNA-FISH was performed with wing imaginal discs on day 6 of control and drug treated fifth instar larvae of *B. mori*. A double-stranded (*ds*) DIG labeled probe 5′-TTAGG-3′ probe (gifted by Frantisek Marec, Director of the Institute of Entomology, Biology Centre of the Academy of Sciences of the Czech Republic) was used. Separated wing discs were rapidly fixed with 4% formaldehyde in PBS for 20 min at room temperature. Fixed wing imaginal disc were incubated in pre-hybridization buffer (40% formamide, 2X SSC, 0.1% SDS, 2 μg/ml salmon sperm DNA) at 60 °C for 1 h. Hybridization was performed at 42 °C overnight in a moist chamber with *ds*-TTAGG probe (1 ng/µl, 30 ng/slide). Anti-DIG FITC (1:300 dilution) was used to visualize the probe. The slides were mounted in Vectasheild with DAPI (Vector Laboratories). The preparations were inspected in a Zeiss Apotome microsope. The FITC and DAPI filters were used to acquire the images.

### Economic parameters measurement

Larvae of both control and drug treated groups were kept undisturbed for three days to accomplish the complete cocoon formation. The cocoon weight was recorded after which it was cut open to obtain the pupal weight and shell weight. The length of silk reeled was also determined. Experiments were done with five rearing of the silkworms. Biological replicates of control and drug treated groups were conducted each time. The data of one biological replicate (control and different drug treated groups) was analysed by one way ANOVA and student’s *t*-test (Two-Sample Assuming Equal Variances). The bars labeled with ‘*’ are significantly different (*p* < 0.05) and labeled with ‘**’ have *p* < 0.01.

### RNA isolation and RT-PCR

Semi-quantitative RT-PCR was performed to substantiate the mRNA expression of broad-complex isoforms and their downstream target genes. The total RNA was isolated from the anterior and posterior part of the silk gland of the control and drug treated worms using TRIzol. The quality of the isolated RNA was checked on a 1% denaturing formaldehyde agarose gel. The total RNA amounting to 5 μg was reverse transcribed using Superscript III and oligo dT primer, essentially following the manufacturer’s protocol. The forward primer BR-C-54 5′-CTTCAACCCGTCTAACTCCTACAACT-3′ was used for RT-PCR of every broad-complex isoform. The reverse primers of the isoforms used were BR-C Z1 5′-GGTCGCATCTGTAATCTTCTTGG-3′, BR-C Z2 5′-GCACAGTACCTTCCCGCATAGT-3′, and BR-C Z4 5′-AGGTGTTGCTGCTCCGTGTG-3′^57^. The forward and the reverse primers of the CPH36 are 5′-CTCATCTCACTCGTGGTTG-3′ and 5′-GGACTTCCTTGATTACAGGC-3′, respectively. The forward and the reverse primers of the CPR151 are 5′-CTCCCTCCCTCGCAAA-3′ and 5′-TACTTTGCTGTTGGGGCTT-3′, respectively. 5′-CCGACGGTAACGAGTCCATT-3′ and 5′-TTGATACGTATGGCCCGCTC-3′ were used as forward and reverse primers for the fibroin gene. A forward primer RP49-51 5′-GGTCAATACTTGATGCCCAACA-3′ and reverse primer RP49-31 5′-GGAATCCATTTGGGAGCATATG-3′ were used for the PCR. From a 20 μL reverse transcription reaction, 1 μL was used directly for PCR amplification of target transcripts using Phusion high fidelity PCR mix (NEB) and different forward/reverse primer sets were used. The annealing temperature was kept at 55 °C for all the primer sets and the amplification was carried out for 35 cycles. The PCR products were analyzed on a 1% TAE agarose gel. Samples were processed in a similar way for developmental gene expression analysis with the posterior silk glands.

### 2D gel and mass spectrometry

On day 6 of the fifth instar larval stage, the silk glands were harvested from control or drug treated (250 μM **DPPA** or 250 µM **TPPA**) larvae and processed to obtain protein extracts for 2D electrophoresis. For total protein isolation, frozen silk gland tissues were grounded in liquid nitrogen and then suspended in lysis buffer (1 mM Tris-HCl, pH 8.0 containing 1 mM PMSF). All further steps were carried out as described earlier^42^. Protein resolution was carried out by iso-electric focusing (IEF) using non-linear pH 3–10, 11 cm IPG strips (Bio-Rad) by cup loading method as per the manufacturer’s protocol (Bio-Rad) followed by 2^nd^ dimensional resolution by 12% SDS-PAGE. The gels were stained with Coommassie Brilliant Blue G250 (CBB) stain. Typically, 300 μg of proteins was applied to IPG strips. Each experiment was repeated at least three times and at least one 2D gel was analyzed in each experiment.

2D gels were imaged using Dyversity-6 gel imager and GeneSnap software (Syngene). PDQuest (version 8.1.0, Bio-Rad) was used to generate a first level match set from three replicate 2-DE gels with a correlation coefficient value of 1.0. Spot detection and between replicate gels were done in automatic detection mode, followed by manual matching to exclude those spots that were not present on all replica gels. The spot densities were normalized using local regression method. Statistical analysis was performed by independent Student t test and the protein spots with p values less than 0.05 were considered as significantly modulated between untreated and drug treated silk glands.

Gel plugs chosen for mass spectrometric analysis were processed for in-gel trypsin digestion essentially following the previously methodology^58, 59^. The protein samples were analyzed by mass spectrometry using UltraFlex III MALDI-TOF/TOF mass spectrometer (Bruker Daltonics). The oligopeptides were co-crystallized with α-cyano-4-hydroxycinnamic acid (5 mg/ml in 0.1% TFA and 50% ACN) on 384-well stain less steel target plate (Bruker Daltonics). Peptide calibration mix I was used to externally calibrate the machine. MALDI-ToF/ToF-MS analysis was performed in positive ion reflection mode. Standard ToF-MS protocol was used to generate mass spectra. Laser was set to fire 150 times per spot. Mass spectra were acquired in the mass range of 600–4500 Da. Peak list was generated using FlexAnalysis software 3.0 (Bruker Daltonics). The mass spectra were exported from FlexAnalysis into the Mascot (Version 2.4.01, Matrix Science) database search engine BioTools 3.1 (Bruker Daltonics). Mascot searches were conducted using the NCBI non-redundant database (release October 2013 with minimum of 32611672 sequences actually searched) with the following settings: Number of miss cleavages permitted was 1; fixed modifications such as carbamidomethyl on cysteine, variable modification of oxidation on methionine residue; peptide tolerance of 100 ppm (or 150 ppm for spot no. 7) enzyme used as trypsin and a peptide charge setting as +1. A match with *D. radiodurans* protein with the best score in each Mascot search was accepted as successful identification. A Mascot score of >70 with a minimum of 8 peptide matches was considered to be a significant identification (*p* < 0.05).

## Electronic supplementary material

Supplementary Info
