# Supplementary material for: Knockdown of Broad-Complex Gene Expression of Bombyx mori by Oligopyrrole Carboxamides Enhances Silk Production
Source: Sci Rep. 2017 Apr 11;7:805. doi: 10.1038/s41598-017-00653-3 (PMC5429751; doi:10.1038/s41598-017-00653-3)
Supplement: Supplementary file 1 — Supplementary Info [file 41598_2017_653_MOESM1_ESM.doc]

**SUPPLEMENTARY INFORMATION**

**Knockdown of Broad-Complex Gene Expression of *Bombyx mori* by Oligopyrrole Carboxamides Enhances Silk Production**

Asfa Ali,^1^ Venugopal Reddy Bovilla,^3^ Danti Kumari Mysarla,^3^ Prasanthi Siripurapu,^3^ Rashmi U. Pathak,^4^ Bhakti Basu,^5^ Anitha Mamillapalli,^3^ Santanu Bhattacharya^1,2*^

**Supplementary Figures**

**E**

**D**

**A**

**B**

**C**

**F**

**Supplementary Figure 1.** Absorption titrations of the drugs with AT-rich and GC-rich sequences. UV-Vis absorption spectral titrations of **(A) DPP** (10 μM) **(B) DPPA** (20 μM) and **(C) TPPA** (20 μM) with increasing concentration of [poly(dA-dT)]_2_ with half-reciprocal plots as inset. Absorption titrations of **(D) DPP** (10 μM) **(E) DPPA** (20 μM) and **(F) TPPA** (20 μM) with increasing concentration of [poly(dG-dC)]_2_. The titrations were performed in sodium phosphate (20 mM), NaCl (20 mM) buffer (pH 7.0) at 25 °C.

**Supplementary Figure 2.** Absorption titration of the drug **DPP** with preformed DNA (*Bm*-dup_20_). Absorption titration of **DPP** (10 μM) in sodium phosphate (20 mM), NaCl (20 mM) buffer (pH 7.0) at 25 °C with increasing concentration of *Bm*-dup_20_ with half-reciprocal plot as inset.

**RP49**

**FIBH**

**5C 5T 7C 7T 9C 9T M**

**BR-C**

**CPH36**


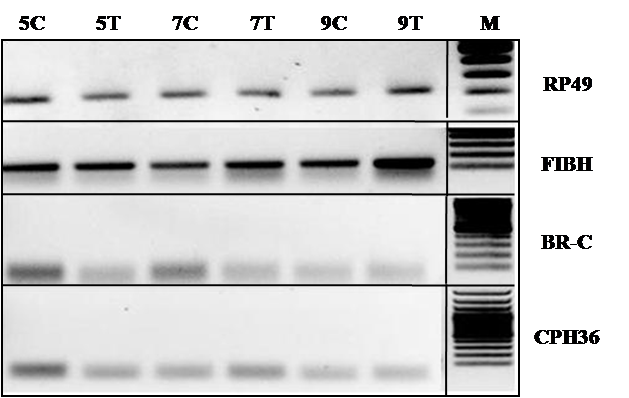


**Supplementary Figure 3.** Time-course RT-PCR data for the expression of RP49, FIBH, broad-complex (BR-C), cuticle gene (CPH36) during day 5, day 7, and day 9 of the fifth instar larval development of the control and the drug treated (**DPP** 250 μM) worms. ‘nC’ and ‘nT’ denote the control and drug treated expression on various days, n (n = 5, 7, 9). ‘M’ denotes the marker lane (100 bp DNA ladder).

.

**Supplementary Figure 4.** ^1^H NMR spectra for **SI-6**.

**Supplementary Figure 5.** ^13^C NMR spectra for **SI-6**.

**Supplementary Figure 6.** ^1^H NMR spectra for **SI-7**.

**Supplementary Figure 7.** ^13^C NMR spectra for **SI-7**.

**Supplementary Figure 8.** ^1^H NMR spectra for **SI-10**.

**Supplementary Figure 9.** ^13^C NMR spectra for **SI-10**.

**Supplementary Figure 10.** ^1^H NMR spectra for **SI-1 (DPP)**.

**Supplementary Figure 11.** ^13^C NMR spectra for **SI-1 (DPP)**.

**Supplementary Figure 12.** ^1^H NMR spectra for **SI-2 (DPPA)**.

**Supplementary Figure 13.** ^13^C NMR spectra for **SI-2 (DPPA)**.

**Supplementary Figure 14.** ^1^H NMR spectra for **SI-11**.

**Supplementary Figure 15.** ^13^C NMR spectra for **SI-11**.

**Supplementary Figure 16.** ^1^H NMR spectra for **SI-3 (TPPA)**.

**Supplementary Figure 17.** ^13^C NMR spectra for **SI-3 (TPPA)**.

**Supplementary Tables**

**Supplementary Table 1**. Binding constant determined from absorption titration.

|  | Binding constant *K*_a_ (10^6^ M^-1^)*^a^* | | |
| --- | --- | --- | --- |
|  | [Poly(dG-dC)]_2_ | [Poly(dA-dT)]_2_ | *Bm*-dup_20_ |
| **DPP** | **-** | **0.3 ± 0.01** | **2.6 ± 0.05** |
| **DPPA** | **-** | **0.2 ± 0.01** | **3.3 ± 0.06** |
| **TPPA** | **-** | **0.1 ± 0.01** | **0.7 ± 0.02** |

*^a^*Absorption titrations were performed with *Bm*-dup_20_, [poly(dA-dT)]_2_, and [poly(dG-dC)]_2_ in a buffer containing sodium phosphate (20 mM), NaCl (20 mM) buffer (pH 7.0) at 25 °C.

**Supplementary Table 2**. Defects witnessed at various stages on treatment with the drugs.

|  | No. of trials | Treated larvae | Complete pupae | Pupae | | Adult-pupae  arrest (%) | Adult | | |
| --- | --- | --- | --- | --- | --- | --- | --- | --- | --- |
|  |  |  |  | Head defect (%) | Eye defect (%) |  | Total | Normal (%) | Wing defect (%) |
| Control | 5 | 25 | 25 | 0 | 0 | 0 | 25 | 100 | 0 |
| **DPP** (100 nM) | 5 | 23 | 23 | 0 | 0 | 0 | 23 | 87 | 13 |
| **DPP** (250 μM) | 4 | 23 | 19 | 8.7 | 8.7 | 21 | 15 | 93.3 | 6.7 |
| **DPPA (**250 μM) | 3 | 19 | 16 | 10.5 | 5.2 | 18.8 | 13 | 84.6 | 15.4 |
| **TPPA** (25 nM) | 3 | 20 | 18 | 10 | 0 | 0 | 18 | 94.5 | 5.5 |
| **TPPA** (250 μM) | 3 | 24 | 22 | 8.3 | 0 | 4.5 | 21 | 85.7 | 14.3 |

**Supplementary Table 3**. BLAST analysis for identification of 5′-TTAGG-3′ repeats in non-telomeric regions.

| Gene | Genomic Region | Match | Exon/Intron |
| --- | --- | --- | --- |
| BR-C gene for broad-complex isoforms Z2, Z4, Z1  (158 kbp with 13exons)  Total gene = 8122-166400 | 8164-8177 | 14/14 (100%) | Exon1  (5′-UTR, proximal transcription start site) |
|  | 128,713-128,704 | 10/10 (100%) | Intron |
|  | 11,794-11,803 | 10/10 (100%) | Intron |
|  | 117,412-117,404 | 9/9 (100%) | Intron |
|  | 144,139-144,131 | 9/9 (100%) | Intron |
|  | 19,426-19,434 | 9/9 (100%) | Intron |
|  | 36,375-36,383 | 9/9 (100%) | Intron |
|  | 63,220-63,228 | 9/9 (100%) | Intron |
|  | 72,630-72,638 | 9/9 (100%) | Intron |
|  | 98,873-98,881 | 9/9 (100%) | Intron |
|  | 112,410-112,418 | 9/9 (100%) | Intron |
|  | 112,947-112,955 | 9/9 (100%) | Intron |
|  | 2165-2172, | 8/8 (100%) | Intron |
|  | 18,411-18,418, | 8/8 (100%) | Intron |
|  | 24,556-24,563 | 8/8 (100%) | Intron |
|  | 45,521-45,514 | 8/8 (100%) | Intron |
|  | 45,541-45,534 | 8/8 (100%) | Intron |
|  | 55,808-55,801 | 8/8 (100%) | Intron |
|  | 70,982-70989 | 8/8 (100%) | Intron |
|  | 91,204-91,211 | 8/8 (100%) | Intron |
|  | 98,874-98,881 | 8/8 (100%) | Intron |
|  | 103,215-103,222 | 8/8 (100%) | Intron |
|  | 112,410-112,417 | 8/8 (100%) | Intron |
|  | 128,713-128,706 | 8/8 (100%) | Intron |
|  | 128,857-128,850 | 8/8 (100%) | Intron |
|  | 138,164-138,157 | 8/8 (100%) | Intron |
|  | 144,138-144,131 | 8/8 (100%) | Intron |
|  | 155,776-155,769 | 8/8 (100%) | Intron |
|  | 162,069-162,076 | 8/8 (100%) | Intron |
|  | 165,972-165,979 | 8/8 (100%) | Exon 13 |
| Putative cuticle protein CPH36 gene | 3722-3733 | 12/12 (100%) | Intron |
| Putative cuticle protein CPR151 gene | 3372-3386 | 15/15 (100%) | Intron |
|  | 3377-3388 | 15/15 (100%) | Intron |
|  | 3371-3381 | 15/15 (100%) | Intron |
| BolA-like 3 (LOC732912), mRNA | 55-67 | 13/13 (100%) | Non-coding region |
| Fibroin heavy chain FIBH gene, complete cds | 4671-4687 | 16/16 (100%) | 5′-promoter region |

**Supplementary Table 4**. Silk gland proteins of the total cellular proteome identified by mass spectrometry.

| Spot. No. | Gene/Locus ID  UniProtKB accession No. | Description | MOWSE Score | Sequence coverage | No. of peptides matched/ searched | Theoretical  MW/pI | Fold change*DPPA/  Control | Fold change*  TPPA/  Control |
| --- | --- | --- | --- | --- | --- | --- | --- | --- |
| 1 | LOC101736010 | Cytosolic non-specific dipeptidase-like | 210 | 48% | 29/77 | 59.3/6.15 | -0.43 | -2.12 |
| 2 | LOC101741707  H9JCV2 | 60 kDa heat shock protein, mitochondrial-like | 157 | 36% | 24/82 | 61.2/5.51 | -0.02 | -1.43 |
| 3 | LOC101741707  H9JCV2 | 60 kDa heat shock protein, mitochondrial-like | 165 | 44% | 28/86 | 61.2/5.51 | -0.07 | -1.83 |
| 4 | LOC692991 | Glycine-tRNA ligase | 199 | 45% | 30/86 | 83.7/7.02 | 0.52 | -6.64 |
| 5 | LOC101735940 | Phosphoribosylformylglycinamidine synthase-like | 215 | 32% | 41/100 | 148.2/5.90 | 0.2 | 2.89 |
| 6 | HM012809  ADQ89808 | Inorganic pyrophosphatase | 163 | 60% | 22/92 | 32.2/4.96 | -1.59 | -0.83 |
| 7 | LOC101745703  H9JUQ4 | Phosphatidylethanolamine-binding protein, isoform X1 | 99 | 36% | 8/60 | 20.3/5.02 | -1.88 | -2.83 |
| 8 | LOC101737545  - | S-formylglutathione hydrolase-like isoform X1 | 123 | 48% | 17/77 | 32.1/5.65 | -0.014 | -2.94 |
| 9 | LOC100101164  Q1HQ47 | NADPH-specific isocitrate dehydrogenase | 198 | 57% | 29/86 | 49.5/8.23 | -1.21 | -6.64 |
| 10 | LOC100101164  Q1HQ47 | NADPH-specific isocitrate dehydrogenase | 248 | 62% | 33/82 | 49.5/8.23 | -6.64 | -6.64 |
| 11 | LOC692848  Q2F645 | Transketolase | 275 | 44% | 27/81 | 67.7/6.4 | 0.09 | -0.014 |
| 12 | LOC692848  Q2F645 | Transketolase | 280 | 46% | 24/55 | 67.7/6.4 | 0.55 | 0.7 |
| 13 | LOC101746890  - | Alanine aminotransferase 2-like | 124 | 38% | 22/73 | 57.5/8.48 | 6.29 | - |
| 14 | LOC100101164  Q1HQ47 | NADPH-specific isocitrate dehydrogenase | 228 | 60% | 30/77 | 49.5/8.23 | 0.58 | - |
| 15 | Jafrac1  Q6T3A7 | Thiol peroxiredoxin | 83 | 45% | 12/62 | 22.1/6.09 | 1.15 | 1.06 |
| 16 | Ter94  Q2V0H5 | Transitional endoplasmic reticulum ATPase TER94 | 135 | 32% | 28/80 | 89.8/5.3 | 0.83 | 2.42 |
| 17 | LOC692786  Q3HR37 | Glyceraldehyde-3-phosphate dehydrogenase | 153 | 53% | 19/75 | 35.5/8.31 | 8.52 | 4.01 |
| 18 | 4EFP_A  - | Lipoprotein 7 | 90 | 46% | 11/42 | 27.5/7.11 | 1.46 | 2.78 |
| 19 | CAA30437  A7LIK7 | Low molecular 30 kDa lipoprotein PBMHPC-23 | 224 | 73% | 24/79 | 30.6/8.46 | 0.07 | 3.02 |
| 20 | CAA38531  Q00801 | Low molecular mass 30 kDa lipoprotein 21G1 | 93 | 46% | 11/68 | 30.3/6.33 | 1.1 | 2.97 |
| 21 | Psat1  Q2F5M8 | Phosphoserine aminotransferase 1 | 183 | 53% | 23/75 | 40.3/6.97 | -1.4 | -2.12 |
| 22 | LOC101743755  - | D-3-phosphoglycerate dehydrogenase-like | 177 | 43% | 20/65 | 35.0/6.54 | 0.124 | -3.32 |
| 23 | LOC692952  Q2F5P8 | Cytosolic malate dehydrogen-ase | 129 | 54% | 19/77 | 35.6/6.85 | 0.137 | -0.058 |
| 24 | LOC101743036  H9JLJ1 | S-adenosylmethionine-dependent methyltransferases | 82 | 42% | 13/54 | 31.4/6.51 | 0.333 | -0.152 |
| 25 | 4IY8_B  - | Lipoprotein_11 | 148 | 67% | 16/67 | 27.7/6.15 | 1.01 | 1.83 |
| 26 | LOC692786  Q3HR37 | Glyceraldehyde-3-phosphate dehydrogenase | 118 | 40% | 13/67 | 35.5/8.31 | -3.32 | -1.6 |
| 27 | LOC692823  Q2F681 | Isocitrate dehydrogenase | 157 | 39% | 21/81 | 46.5/6.24 | -0.34 | -2.47 |
| 28 | AK  Q2F5T5 | Arginine kinase | 177 | 57% | 19/75 | 40.3/5.87 | 0.926 | -6.64 |
| 30 | LOC100037426  A1YQ87 | Enolase | 166 | 43% | 20/71 | 47.2/5.62 | 0.014 | 0.084 |
| 31 | LOC100101173  Q1HPU7 | S-adenosyl-L-homocysteine hydrolase | 100 | 26% | 16/75 | 47.9/5.79 | -0.014 | 0.65 |
| 32 | AF315317_1  - | Heat shock protein hsp20.8 | 107 | 49% | 11/71 | 20.8/5.98 | -1.12 | -1.36 |
| 33 | Atpsyn-beta  Q1HPT1 | H+ transporting ATP synthase beta subunit isoform 2 | 105 | 34% | 15/64 | 54.8/5.32 | -1.21 | -1.152 |
| 34 | AGR44790  P84183 | Actin-4 | 162 | 41% | 17/63 | 42.1/5.22 | 0.55 | -2.18 |
| 35 | A2  P07837 | Actin, muscle-type A2 | 155 | 49% | 18/78 | 42.2/5.29 | -1.09 | 0.613 |
| 36 | AGR44834  P84183 | Actin-4 | 147 | 46% | 16/61 | 42.1/5.22 | -0.3 | -1.6 |

* Fold change is represented as log_2_ values. Red ≥ 1.0 fold, blue ≤ 1.0 fold and black value intermediate of ± 1.

**Supplementary Methods**

**General.** NMR spectra were recorded using a Bruker AMX NMR spectrometer (400 MHz for ^1^H NMR and 100 MHz for ^13^C NMR). The following abbreviations were used to illustrate the ^1^H NMR multiplicities: s = singlet, br s = broad singlet, d = doublet, t = triplet, q = quartet, m = multiplet. Mass spectra were recorded on a Micromass Q-TOF Micro TM spectrometer. IR spectra were recorded on FT-IR Perkin Elmer Spectrum GX spectrometer. Analytical thin layer chromatography (TLC) used to monitor reactions were carried out on silica gel plates (60F-254) from Merck followed by visualization in UV and iodine chamber. Purification of crude products were performed based on column chromatography using silica gel (mesh: 60-120 and 100-200) and aluminium oxide (neutral) from Fisher Scientific.

All starting materials (chemicals) were from the best known sources and used as purchased. All solvents were obtained from Merck and if required, were distilled and/or dried prior to use. All the ligands were >95% pure as confirmed by elemental analysis. Stock solution of the drugs were prepared in DMSO and diluted in required buffer prior or water to use. HPLC purified oligonucleotides [poly(dG-dC)]_2_, [poly(dA-dT)]_2_, d(TTAGG)_4_, and d(CCTAA)_4_ were purchased from Sigma, Genosys, Bangalore. The purity of the oligonucleotides was confirmed using high resolution sequencing gel. The concentrations of the DNA were determined spectrophotometrically and expressed as base-pairs with *ε* values (M^-1^ cm^-1^): [poly(dA-dT)]_2_; *ε*_262_ = 13200 and [poly(dG-dC)]_2_; *ε*_262_ = 16800. The concentration of each oligonucleotide was determined from absorbance measurements at 260 nm based on their molar extinction coefficients (*ε*_260_) 228900 and 219950, respectively, for d(TTAGG)_4_ and d(CCTAA)_4_. *B. mori* (BV CSR2 × CSR4) strains were procured from the Department of Sericulture, Government of Andhra Pradesh. The larvae were reared in cardboard boxes at 23 ± 1 °C and 65–70% relative humidity. They were fed with fresh mulberry leaves until the fifth instar stage. TRIzol required for RNA isolation and Superscript III used for reverse transcription were purchased from Invitrogen. Trypsin sequencing grade, modified for proteomics was purchased from Roche (Cat. No. 11418025001). All the chemicals for running 2D gels including IEF strips were purchased from BioRad.

**Scheme S1:** Synthesis of the drugs (**DPP**, **DPPA** and **TPPA**)

**SI-5 (2-Chloro-N-[6-(2-chloroacetamido)pyridin-2-yl] acetamide)**: 2, 6-Diaminopyridine (**SI-4**) (5.0 g, 45.9 mmol, 1 equiv.) and N, N-diisopropylethylamine (DIPEA) (20 mL, 114.8 mmol, 2.5 equiv.) were taken in dry THF (30 mL) and kept at 0 °C, to which a solution of chloroacetyl chloride (8.0 mL, 101.0 mmol, 2.2 equiv.) in dry THF (15 mL) was added dropwise, maintaining the temperature at 0 °C. The reaction mixture was heated at 60 °C for a period of 12 h, after which the THF was removed under reduced pressure. Water (20 mL) was added and the crude product was extracted with ethyl acetate (3×50 mL) and purified by flash chromatography over silica gel (mesh 60-120, 0:100 to 0.5:95.5 MeOH : CHCl_3_) to yield an off-white solid. Spectral (NMR, mass and IR) data are in agreement with the published report^1^.

**SI-6 (2-Azido-N-[6-(2-azidoacetamido)pyridin-2-yl] acetamide)**: Compound **SI-5** (7.0 g, 26.7 mmol, 1 equiv.) was dissolved in dry DMF (12 mL) to which sodium azide (17.4 g, 267.0 mmol, 10 equiv.) was added and the mixture was heated at 60 °C in inert atmosphere for 16 h. The solvent was removed in vacuum, followed by the addition of water (10 mL) and extraction with ethyl acetate (3×50 mL). The crude solid thus obtained was purified by column chromatography over silica gel (mesh 60-120, 1:99 MeOH:CHCl_3_) resulting in a pure white solid (4.78 g, 65%). Analytical data: TLC (2:98 MeOH:CHCl_3_) *R_f_* = 0.45; ^1^H NMR (400 MHz, *d_6_*-DMSO): δ 10.33 (s, 2H), 7.82-7.74 (m, 3H), 4.14 (s, 4H); ^13^C NMR (100 MHz, *d_6_*-DMSO): δ 167.41, 149.78, 140.52, 109.39, 51.38; HRMS (*m*/z): calcd. for C_9_H_9_N_9_O_2_, [M+Na]^+^, 298.0777; found, 298.0778; IR (KBr, cm^-1^) 3336, 2108, 1684, 1529, 1448, 1297, 1244, 1157, 1098, 791, 669. Anal. (C_9_H_9_N_9_O_2_) calcd: C 39.28, H 3.30, N 45.80; found: C 39.21, H 3.31, N 45.92.

**SI-7 (2-Amino-N-[6-(2-aminoacetamido)pyridin-2-yl] acetamide)**: Compound **SI-6** (4.0 g, 14.5 mmol, 1 equiv.) dissolved in methanol was reduced over H_2_-Pd/C (1.3 g, 10%) under 4 atm pressure for 24 h. TLC was checked to ensure complete conversion of the azide to amine. The reaction mixture was then filtered through a bed of celite and the solvent was quickly removed under reduced pressure (2.43 g, 75%). Analytical data: TLC (10:90 MeOH:CHCl_3_) *R_f_* = 0.15; ^1^H NMR (400 MHz, *d_6_*-DMSO): δ 7.85-7.77 (m, 3H), 3.79 (s, 4H); ^13^C NMR (100 MHz, *d_6_*-DMSO): δ 166.74, 149.68, 140.69, 109.36, 41.63; HRMS (*m*/z): calcd. for C_9_H_13_N_5_O_2_, [M+H]^+^, 224.1147; found, 224.1146; IR (KBr, cm^-1^) 3444, 1600, 1385, 1352, 1120, 742, 695. Anal. (C_9_H_13_N_5_O_2_) calcd: C 48.42, H 5.87, N 31.37; found: C 48.26, H 5.86, N 31.43.

**SI-10 ({5-[5-{[({6-[2-({4-[4-(hydroxynitroso)-1-methyl-1H-pyrrole-2-amido]-1-methyl-1H-pyrrol-2-yl}formamido)acetamido]pyridin-2-yl}carbamoyl)methyl]carbamoyl}-1-methyl-1H-pyrrol-3-yl)carbamoyl]-1-methyl-1H-pyrrol-3-yl} azinic acid)**: 4-(1-methyl-4-nitro-1H-pyrrole-2-carboxamido)-1-methyl-1H-pyrrole-2-carboxylic acid **SI-8**^2^ (0.964 g, 3.3 mmol, 2.2 equiv.) was dissolved in dry DMF (10 mL) and was cooled to 0 °C. To the above solution, N, N′-dicyclohexylcarbodiimide (0.886 g, 4.3 mmol, 2.9 equiv.) and N-hydroxysuccinimide (0.575 g, 5.0 mmol, 3.3 equiv.) were added and the resulting solution was stirred at 0 °C for 1 h and then at r.t. for another 6 h. Freshly prepared 2,6-bis-(2-amino-acetylamino)-pyridine **SI-7** (0.334 g, 1.5 mmol, 1 equiv.) was added and stirring was continued for 2.5 h at rt. Ethyl acetate (15 mL) was added leading to precipitation which was washed successively with 5% NaHCO_3_ and brine. The ethyl acetate layer was kept overnight leading to the crystallization of yellow solid product (0.462 g, 40%). Analytical data: TLC (2:98 MeOH:CHCl_3_) *R_f_* = 0.5; ^1^H NMR (400 MHz, *d_6_*-DMSO): δ 10.42 (s, 2H), 10.30 (s, 1H), 8.44 (t, *J* = 5.6 Hz, 1H), 8.19 (s, 2H), 8.17 (s, 1H), 7.73 (s, 3H), 7.59-7.57 (m, 3H), 7.28 (s, 1H), 7.19 (d, *J* = 1.6 Hz, 2H), 6.98 (s, 1H), 3.96 (s, 8H), 3.87 (s, 6H), 3.82 (s, 2H); ^13^C NMR (100 MHz, *d_6_*-DMSO): δ 170.77, 157.26, 155.44, 133.92, 133.87, 128.50, 125.94, 124.46, 123.26, 113.25, 110.35, 107.94, 37.51, 36.36, 25.53; HRMS **(*m*/**z**)**: calcd. for C_33_H_33_N_13_O_10_ [M+Na]^+^, 794.2371; Found, 794.2369; IR (KBr, cm^-1^) 3418, 3139, 2926, 2852, 1762, 1732, 1652, 1584, 1506, 1446, 1418, 1304, 1204, 1116, 1081, 990, 976, 813. Anal. (C_33_H_33_N_13_O_10_) calcd: C 51.36, H 4.31, N 23.60; found: C 51.50, H 4.33, N 23.53.

**SI-1 (DPP) (4-Formamido-N-{5-[({[6-(2-{[4-(4-formamido-1-methyl-1H-pyrrole-2-amido)-1-methyl-1H-pyrrol-2-yl]formamido}acetamido)pyridin-2yl]carbamoyl}methyl)carbamoyl] -1-methyl-1H-pyrrol-3-yl}-1-methyl-1H-pyrrole-2-carboxamide)**: Compound **SI-10** (0.200 g, 0.26 mmol, 1 equiv.) was dissolved in dry DMF (3 mL) and reduced using H_2_-Pd/C (0.540 g, 10%) for 24 h at rt. The resultant mixture was passed through a bed of celite and formic acid (0.03 mL, 0.78 mmol, 3 equiv.) was added to the resultant filtrate followed by the addition of N, N′-dicyclohexylcarbodiimide (0.160 g, 0.78 mmol, 3 equiv.) at 0 °C under nitrogen atmosphere. The solution was first stirred at 0 °C for 1 h and then the stirring was continued for 24 h at rt. The solvent was removed under reduced pressure and ethyl acetate (5 mL) was added and washed successively with 5% NaHCO_3_ to yield a light brown solid which was dried in vacuum (0.16 g, 82%). Analytical data: TLC (20:80 MeOH:CHCl_3_) *R_f_* = 0.25; ^1^H NMR (400 MHz, *d_6_*-DMSO): δ 10.08 (s, 1H), 9.99 (s, 2H), 9.95 (s, 1H), 8.40 (s, 2H), 8.13 (s, 1H), 7.77 (s, 3H), 7.25 (s, 2H), 7.20 (s, 1H), 7.07 (s, 1H), 6.98 (s, 2H), 6.92 (s, 1H), 4.02 (s, 4H), 3.86 (s, 3H), 3.84 (s, 3H), 3.81 (s, 6H); ^13^C NMR (100 MHz, *d_6_*-DMSO): δ 168.97, 161.60, 158.33, 157.89, 150.04, 122.22, 122.09, 120.73, 118.50, 118.42, 108.68, 104.78, 104.00, 36.07, 24.43; HRMS (*m*/z): calcd. for C_35_H_37_N_13_O_8_[M+Na]^+^, 790.2786; found, 790.2788; IR (neat, cm^-1^) 3674, 3266, 3109, 1677, 1567, 1447, 1400, 1290, 1188, 1091, 803, 781, 733, 717. Anal. (C_35_H_37_N_13_O_8_) calcd: C 54.75, H 4.86, N 23.72; found: C 54.56, H 4.88, N 23.65.

**SI-2 (DPPA) (4-Amino-N-{5-[({[6-(2-{[4-(4-amino-1-methyl-1H-pyrrole-2-amido)-1-methyl-1H-pyrrol-2-yl]formamido}acetamido)pyridin-2-yl]carbamoyl}methyl)carbamoyl]-1-methyl-1H-pyrrol-3-yl}-1-methyl-1H-pyrrole-2-carboxamide)**: Compound **SI-10** (0.200 g, 0.26 mmol, 1 equiv.) was reduced over H_2_-Pd/C (0.540 g, 10%) for 24 h at rt which upon filtration was concentrated under vacuum followed by the addition of 1 N HCl to yield a white solid which was further dried (0.171 g, 80%). Analytical data: TLC (20:80 MeOH:CHCl_3_) *R_f_* = 0.2; ^1^H NMR (400 MHz, *d_6_*-DMSO): δ 10.33 (s, 4H), 10.20 (s, 1H), 10.04 (s, 1H), 10.01 (s, 1H), 8.47 (br s, 1H), 8.38 (s, 1H), 7.74 (s, 2H), 7.27 (s, 2H), 7.11-7.07 (m, 4H), 7.01 (s, 1H), 4.02 (s, 3H), 3.88 (s, 3H), 3.85 (s, 2H), 3.83 (s, 2H), 3.81 (s, 6H); ^13^C NMR (100 MHz, *d_6_*-DMSO): δ 173.92, 173.20, 158.06, 124.98, 122.44, 121.94, 120.73, 119.95, 113.21, 108.83, 107.57, 36.78, 36.42, 25.44; HRMS (*m*/z): calcd. for C_33_H_37_N_13_O_6_ [M+H]^+^, 712.3069; found, 712.3068; IR (neat, cm^-1^) 3402, 2926, 2852, 2658, 2089, 1633, 1393, 1088, 1017, 913, 774. Anal. (C_33_H_37_N_13_O_6_.2HCl) calcd: C 50.51, H 5.01, N 23.21; found: C 50.34, H 5.03, N 23.29.

**SI-11 ({5-[(5-{[5-({[(6-{2-[(4-{4-[4-(Hydroxynitroso)-1-methyl-1H-pyrrole-2-amido]-1-methyl-1H-pyrrole-2-amido}-1-methyl-1H-pyrrol-2-yl)formamido]acetamido}pyridin-2-yl) carbamoyl]methyl}carbamoyl)-1-methyl-1H-pyrrol-3-yl]carbamoyl}-1-methyl-1H-pyrrol-3-yl)carbamoyl]-1-methyl-1H-pyrrol-3-yl} azinic acid)**: 1-Methyl-4-[1-methyl-4-(1-methyl-4-nitropyrrole-2carboxamido]-pyrrole-2-carboxylic acid **SI-9**^3^ (1.532 g, 3.7 mmol, 2.2 equiv.) was dissolved in dry DMF (10 mL) at 0 °C and to it, N, N′-dicyclohexylcarbodiimide (1.009 g, 4.9 mmol, 2.9 equiv.) and N-hydroxysuccinimide (0.644 g, 5.6 mmol, 3.3 equiv.) were added and the resulting solution was stirred at 0 °C for 1 h and then at r.t. for another 6 h. Freshly prepared 2,6-bis-(2-amino-acetylamino)-pyridine **SI-7** (0.379 g, 1.7 mmol, 1 equiv.) was added and stirring was continued for 2.5 h at rt. Ethyl acetate (16 mL) was added leading to precipitation which was washed successively with 5% NaHCO_3_ and brine. The ethyl acetate layer was kept overnight leading to the crystallization of yellow solid product (0.726 g, 42%). Analytical data: TLC (2:98 MeOH:CHCl_3_) *R_f_* = 0.45; ^1^H NMR (400 MHz, *d_6_*-DMSO) δ 10.39 (s, 1H), 10.36 (s, 1H), 10.17 (s, 1H), 10.01 (s, 1H), 9.97 (s, 1H), 8.41 (t, *J* = 5.2 Hz, 1H), 8.30 (s, 1H), 8.16 (s, 2H), 7.75 (s, 3H), 7.62 (s, 2H), 7.28 (s, 2H), 7.26 (s, 1H), 7.23 (s, 1H), 7.14 (s, 1H), 7.08 (s, 1H), 7.00 (s, 1H), 4.03 (s, 3H), 3.96 (s, 6H), 3.86 (s, 9H), 3.81 (s, 4H); ^13^C NMR (100 MHz, *d_6_*-DMSO) δ 170.78, 161.69, 158.52, 157.04, 157.02, 156.98, 133.88, 128.21, 126.37, 126.34, 124.46, 124.06, 123.05, 122.58, 122.33, 122.18, 121.65, 121.44, 112.92, 110.43, 107.72, 37.51, 36.26, 36.23, 36.19, 36.13, 24.48; HRMS (*m*/z): calcd. for C_45_H_45_N_17_O_12_ [M+Na]^+^, 1038.3331; found, 1038.3333; IR (neat, cm^-1^) 3431, 3132, 2934, 2853, 1765, 1724, 1652, 1430, 1403, 1312, 1213, 1087, 815. Anal. (C_45_H_45_N_17_O_12_) calcd: C 53.20, H 4.46, N 23.44; found: C 53.09, H 4.48, N 23.37.

**SI-3 (TPPA) (4-(4-Amino-1-methyl-1H-pyrrole-2-amido)-N-(5-{[({6-[2-({4-[4-(4-amino-1-methyl-1H-pyrrole-2-amido)1-methyl-1H-pyrrole-2-amido]-1-methyl-1H-pyrrol-2-yl} formamido)acetamido]pyridin-2-yl}carbamoyl)methyl]carbamoyl}-1-methyl-1H-pyrrol-3-yl)-1-methyl-1H-pyrrole-2-carboxamide)**: Compound **SI-11** (0.250 g, 0.25 mmol, 1 equiv.) was reduced over H_2_-Pd/C (0.68 g, 10%) for 24 h at rt which upon filtration was further concentrated in vacuum followed by the addition of 1 N HCl to yield a white solid which was dried in vacuum (0.213 g, 81%). Analytical data: TLC (20:80 MeOH:CHCl_3_) *R_f_* = 0.2; ^1^H NMR (400 MHz, *d_6_*-DMSO) δ 10.33 (s, 5H), 10.23-10.20 (m, 2H), 10.12 (s, 1H), 10.04 (s, 1H), 8.48 (br s, 1H), 7.79 (s, 1H), 7.73 (s, 1H), 7.60 (s, 1H), 7.47 (s, 1H), 7.35 (s, 1H), 7.29-7.24 (m, 4H), 7.13-7.06 (m, 6H), 7.02 (s, 1H), 3.91 (s, 9H), 3.87 (s, 13H); ^13^C NMR (100 MHz, *d_6_*-DMSO) δ 173.55, 173.19, 172.80 169.16, 157.81, 156.86, 124.78, 122.82, 122.36, 122.34, 122.05, 121.87, 121.64, 118.64, 115.85, 113.13, 111.83, 108.89, 108.80, 107.43, 104.92, 36.60, 36.13, 35.85, 24.42; MALDI (*m*/z): calcd. for C_45_H_49_N_17_O_8_ [M+Na]^+^, 978.384; found, 978.380; IR (neat, cm^-1^) 3423, 2255, 2128, 1656, 1449, 1051, 1026, 1006, 825, 764. Anal. (C_45_H_49_N_17_O_8_.2HCl) calcd: C 52.53, H 5.00, N 23.14; found: C 52.39, H 4.99, N 23.21.

**References**

1. Harte, A. J. & Gunnlaugsson, T. Synthesis of α-chloroamides in water. *Tetrahedron Lett.* **47**, 6321-6324 (2006).
2. Lown, J. W. & Krowicki, K. Efficient total syntheses of the oligopeptide antibiotics netropsin and distamycin. *J. Org. Chem.* **50**, 3774-3779 (1985).
3. Bialer, M., Yagen, B. & Mechoulam, R. A total synthesis of distamycin a, an antiviral antibiotic. *Tetrahedron* **34**, 2389-2391 (1978).
